# Our Daughters

**Published:** 1889-11

**Authors:** Mary A. Livermore


### OUR DAUGHTERS.

All women should have an early training commensurate with the greatness of the work that they only can perform. Let our young daughters be garnished with accomplishments, if you will. Let them have amusements, and live and breathe in a sunny, gay atmosphere. Encourage them to cultivate that habit of looking at the best and brightest side of things, which Dr. Johnson has pronounced "worth a thousand pounds a year." Do not repress their girlish enthusiasm over their pursuits or their pleasures. They will have need of a large store before they are done with life. Give to them the highest education demanded by the hunger of their souls, and allow them to fit for any calling or profession to which they are adapted by their tastes and capacities.

But by no means neglect what Canon Kingsley calls their "lower education." Let them have an acquaintance with themselves, with their own physiology, and the laws controlling it. Let them be trained, as far as possible, as if you were sure they were to be wives, mothers and housekeepers, even when they receive in addition, technical training. But few women reach adult life, even when they do not marry, without finding themselves so circumstanced at times that a domestic training is invaluable. Thus will our daughters be prepared to do better work in the world, to rear nobler children. Trained and self-poised, they will not be in bondage to ignorance; nor will they be as liable to become the dupes or the prey of those human sharks who are ever on the alert to lead astray unwary girls.—*Mrs. Mary A. Livermore.*

